# Density-dependent adjustment of inducible defenses

**DOI:** 10.1038/srep12736

**Published:** 2015-08-03

**Authors:** Ralph Tollrian, Sonja Duggen, Linda C. Weiss, Christian Laforsch, Michael Kopp

**Affiliations:** 1 Department of Animal Ecology, Evolution and Biodiversity, Ruhr-University Bochum, Germany; 2 Animal Ecology I and BayCEER, University of Bayreuth, Germany; 3 Section of Evolutionary Ecology, Department Biology II, Ludwig-Maximilian-University Munich, Germany; 4 Department of Physiological Ecology, Max-Planck-Institute of Limnology, Plön, Germany

## Abstract

Predation is a major factor driving evolution, and organisms have evolved adaptations increasing their survival chances. However, most defenses incur trade-offs between benefits and costs. Many organisms save costs by employing inducible defenses as responses to fluctuating predation risk. The level of defense often increases with predator densities. However, individual predation risk should not only depend on predator density but also on the density of conspecifics. If the predator has a saturating functional response one would predict a negative correlation between prey density and individual predation risk and hence defense expression. Here, we tested this hypothesis using six model systems, covering a taxonomic range from protozoa to rotifers and crustaceans. In all six systems, we found that the level of defense expression increased with predator density but decreased with prey density. In one of our systems, i.e. in *Daphnia*, we further show that the response to prey density is triggered by a chemical cue released by conspecifics and congeners. Our results indicate that organisms adjust the degree of defense to the acute predation risk, rather than merely to predators’ densities. Our study suggests that density-dependent defense expression reflects accurate predation-risk assessment and is a general principle in many inducible-defense systems.

Predation is a major factor driving evolution, and the ability to defend against attackers is a key factor for the evolutionary success of most living organisms. As many defenses incur trade-offs and evolve in a cost-benefit framework, organisms are frequently selected to express only the level of defense that is necessary under the specific environmental conditions. Consequently, inducible defenses, which allow for flexible adjustment of behavioral, physiological or morphological adaptations, have been found to be widespread^1^. To avoid fitness losses resulting from over- or under-expression of defenses, prey face the problem to accurately assess their current predation risk. In recent years, many species have been shown to adjust their level of defense to the density of predators^1^,^2^. However, the individual predation risk does not only depend on the predator’s density but also on the density of prey. A major reason is the dilution effect (‘safety in numbers’), which also plays a key role in the evolution of grouping behavior^3^,^4^. The dilution effect is present whenever the number of prey a predator consumes per unit time is a saturating or decreasing function of prey density (e.g., if the functional response is of type II), and the number of predators is constant over the time-scale of interest (i.e., a high prey density does not attract relatively more predators). If these conditions are met, and if the defense is subject to a trade-off, prey should reduce their investment in defenses if their own density is high^5^,^6^,^7^. A similar response should also result if high prey density increases the costs of the defense, for example, when it correlates with resource depletion^8^,^9^,^10^,^11^. Such density-dependent benefits for prey have rarely been considered in studies of inducible defenses. Wiackowski & Starońska^12^ found first evidence in experiments with ciliates but could not rule out that the results were influenced by food effects; Peacor^5^ developed a theoretical model suggesting that these effects should occur; McCoy^13^ and Van Buskirk *et al*.^14^ found prey density effects on defense expression in amphibian tadpoles. However, so far it is not known whether prey density adjustment of defenses can be found in many taxa. Here, we tested the hypothesis that prey density influences the expression of inducible defenses in a wide range of organisms, in a study using six predator-prey systems with protozoan, rotifer and crustacean prey. In all six systems, this hypothesis was confirmed indicating that prey density-dependent adjustment of inducible defenses is a general principle.

## Results

### Experiment 1: Effect of predator and prey density on defense expression

**Figure 1:**
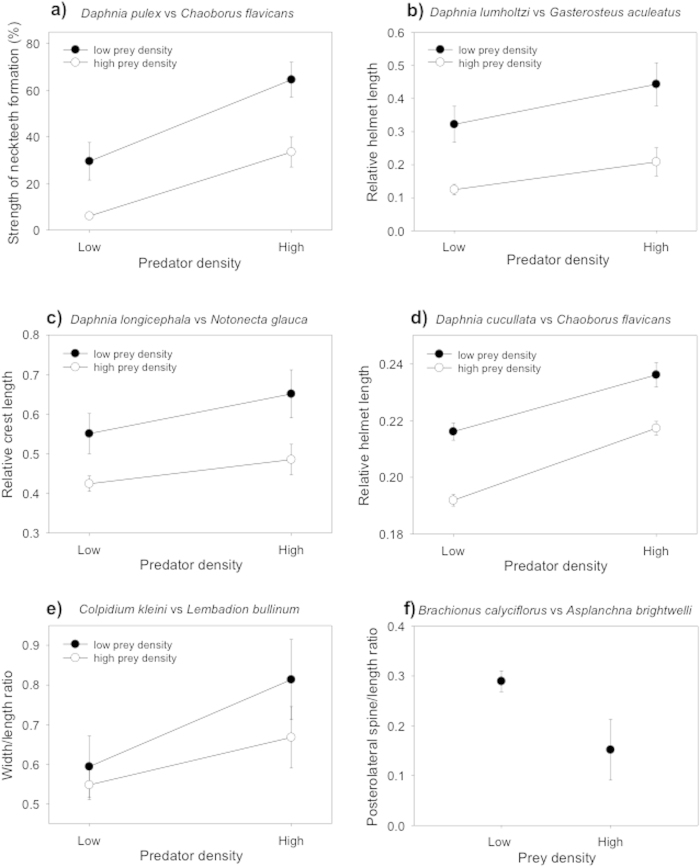
Expression of inducible defenses increases with predator density (kairomone concentration) and decreases with prey density. The plots show treatment means ± SD of relative defense expression (trait value divided by body length) or, in *Daphnia pulex*, of a score of neckteeth formation. Two-way ANOVA results are significant for predator and prey effects in all systems (all *p* ≤ 0.001). In the *Brachionus calyciflorus* system, the prey density effect was tested only at a single predator density and analyzed with a Mann-Whitney U-test (*p* = 0.023).

In all six predator-prey systems, the degree of defense formation increased with predator density, but decreased with prey (i.e., conspecifics) density (Fig. 1). The effect of prey density was present at both, low and high, predator densities. Two-way ANOVA results are significant for predator and prey effects in all systems (Fig. 1a–e; a) *D. pulex*: predator *p* = 0.001, prey *p* < 0.001, interaction *p* = 0.563; b) *D. lumholtzi*: predator *p* < 0.001, prey *p* < 0.001, interaction *p* = 0.040; c) *D. longicephala*: predator *p* < 0.001, prey *p* < 0.001, interaction *p* = 0.791; d) *D. cucullata*: predator *p* < 0.001, prey *p* < 0.001, interaction *p* = 0.261; e) *C. kleini*: predator *p* < 0.001, prey *p* < 0.001, interaction *p* < 0.01). In the *Brachionus calyciflorus* system (Fig. 1f), the prey-density effect was tested only at a single predator density and was significant according to a Mann-Whitney U-test (*p* = 0.023). Thus, our study suggests that organisms are able to assess their individual predation risk based not only on predator density but also on prey density and to adjust their defenses accordingly.

### Experiment 2a: Nature of the cue used by *D. lumholtzi*

**Figure 2:**
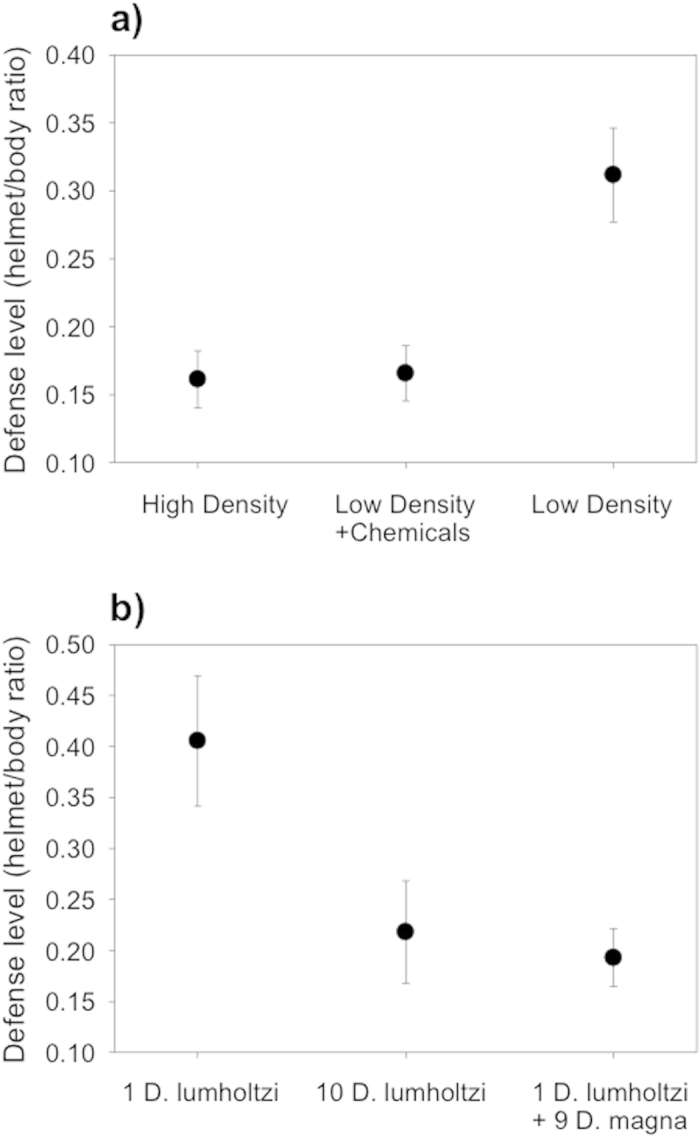
Nature and specificity of the cue for the prey-density effect in *Daphnia lumholtzi*. (**a**) Information about prey density is transmitted via a chemical cue. Medium from conspecifics in a high density added to a low prey density reduced the induction to a level not significantly different from that in the high-density treatment (Tamhane pairwise comparison, *p* = 0.921). The low prey-density treatment differs significantly from both other treatments (Tamhane pairwise comparison, all *p* < 0.001). (**b**) The chemical cue is not species specific. One *D. lumholtzi* reared together with 9 *D. magna* reduced the defense induction to a level not significantly different from the high-density treatment (10 conspecifics, Tamhane pairwise comparison, *p* = 0.545). The low-density treatment differed significantly from both other treatments (Tamhane pairwise comparison, all *p* < 0.001). Shown are treatment means ± SD.

When reared at low density but in medium transferred from a high-density culture, *D. lumholtzi* showed a level of defense similar to the one observed in the high-density control (Tamhane pairwise comparison, *p* = 0.921) and significantly weaker than in the low-density control (Tamhane pairwise comparison, *p* < 0.001). This shows that the daphniids measure conspecifics density via a chemical cue (Fig. 2a).

### Experiment 2b: Species-specificity of the cue used by *D. lumholtzi*

When reared at low density together with a high density of *D. magna*, *D. lumholtzi* expressed a level of defense similar to the one observed at high conspecifics density (Tamhane pairwise comparison *p* = 0.545). In both of these treatments, the response was significantly weaker than at low conspecifics density without *D. magna* (Tamhane pairwise comparison, all *p* < 0.001). Thus, *D. lumholtzi* reacted to chemical cues from *D. magna*, showing that these cues are not species-specific (Fig. 2b).

### Discussion

We have shown that prey organisms from six predator-prey systems reduce the expression of morphological inducible defenses if their own population density is high. Similar results have also been reported in earlier studies of the ciliate *Euplotes octocarinatus*^12^ and amphibian tadpoles of *Hyla chrysoscelis*^13^ and *Rana temporaria*^14^. Together with these findings, the wide taxonomic range of our study organisms (covering protozoa, rotifers and crustaceans) suggests a general principle: Prey use information about the density of both predators and conspecifics to determine their investment in inducible defenses.

In our *Daphnia lumholtzi* system, we could further show that the response to prey density is triggered by a chemical cue released from conspecifics or congeners. In treatments with prey-specific chemical cues, mimicking a high prey density, the same density-dependent reduction of defenses was observed even under actually low prey densities. Thus, this response is independent of individual food supply.

It is possible that this cue is similar to the so-called ‘crowding chemical’, which at very high concentrations leads to life-history shifts and induces resting-egg production in *Daphnia*^15^. Nevertheless, none of these responses was observed in our experiments, probably because *Daphnia* densities were still relatively low.

What are the ultimate reasons for these prey density-dependent modifications of defense expression? – With respect to the usual cost-benefit framework, our results suggest that prey density either increases the costs of the defense, decreases the benefit, or both. Distinguishing between these alternatives requires detailed quantitative knowledge about costs and benefits at various prey densities, which is generally not available. In the following, we will discuss our results in the light of published studies.

Density-dependent benefits for prey have rarely been considered in studies of inducible defenses (but see^5^,^12^,^13^,^14^), yet our data suggest that they are widespread. Since most predators (including those used in our study) have type II functional responses^16^, which are saturating over the whole range of prey densities, this scenario is most likely a common phenomenon in nature. Furthermore, even type I functional responses^17^ enter a saturating phase once prey density exceeds a threshold. Thus, measuring conspecifics’ density can be seen as a part of the prey’s predation-risk assessment^2^,^12^,^13^,^18^.

Quantitative evidence for a density-dependent defense benefit can be obtained from the study by Jeschke and Tollrian^18^, who measured functional responses of the phantom midge larvae *Chaoborus flavicans* (a relative of *C. obscuripes* used here) feeding on defended and undefended *Daphnia pulex*. They found that the individual predation risk of undefended daphnids decreased by more than 50% over the range of daphnid densities used in the present study, thus demonstrating the dilution effect. In addition, high densities reduced the relative advantage of defended over undefended daphnids (i.e., the ratio of survival probabilities), that is, the benefit of the defense was indeed density-dependent.

A model by Peacor (2003)^5^ also predicts density-dependent reduction of defenses at high prey-densities, but for a slightly different reason than the one proposed here. The model considers situations where prey detect predators via alarm cues from injured or killed conspecifics (instead of predator kairomones). Here, the cue concentration is proportional to the number of actual predation events, and the individual predation risk associated with a given cue concentration decreases with prey density even if the predator’s functional response is linear (i.e., in the absence of the dilution effect). Prey should then measure conspecifics density to correct for this effect. This mechanism might have some relevance in *Daphnia*, where alarm cues have been shown to contribute to defense induction (along with predator kairomones^19^,^20^), but cannot have played a role in our study, because we only used kairomones. The difference between the dilution effect and the alarm-cue effect has not always been clearly appreciated. For example, McCoy (2007)^13^ purported to test Peacor’s model, but used caged predators (releasing kairomones, not alarm-cues) and discussed his results in terms of the dilution effect.

It is worth noting that, if a high prey density attracts (or raises) a high number of attackers, individual predation risk may actually increase with conspecifics density. This is likely to be the case in plant-herbivore or host-parasite systems, where the risk of infestation often increases with density. Accordingly, several insect species have been shown to increase their investment in immunity under crowded conditions (so-called ‘density-dependent prophylaxis’^21^,^22^).

Similarly, non-linear effects of predator density might have influenced the interaction effects in our study. We changed prey and predator density with the factor of ten. Thus, we could assume that the effect of a ten-fold higher predator density on individual predation risk is compensated by a ten-fold higher conspecific density. Indeed we found in 3 systems no significant interaction effects. In two systems however the interaction effects were significant. A possible explanation is that an increase in predator density is affecting predation risk in a nonlinear fashion.

Alternatively or in addition to being related to predation-risk assessment (and therefore, density-dependent benefits), reduced defense expression at high prey density might also be a consequence of density-dependent costs^10^. For example, induced defended *Daphnia* have increased vulnerability to parasites^23^, and the associated disadvantage is likely to be more severe under crowded conditions, where infection risk is higher.

In many cases, costs are likely to increase with prey density if high density correlates with resource depletion. This seems particularly likely for allocation costs^24^ or costs that reduce foraging efficiency and, hence, competitive ability^9^,^25^,^26^ (see below). Accordingly, several studies have shown that direct measures of costs are larger under low-food than under high-food conditions (e.g.^8^,^9^,^10^,^11^ or that organisms reduce their investment in defense under food limitation^8^,^27^,^28^,^29^,^30^). In our experiments, we aimed to exclude potential effects of resource depletion by providing unlimited food (or no food at all). This alone, however, does not rule out density-dependent costs as ultimate reason for our results, because prey perceiving a high conspecifics density might anticipate resource depletion in the future. How likely is this scenario? – In *Colpidium kleini*, defended cells had their growth rate reduced by about 25% at high food concentration and by about 50% at low food concentration^11^, which might suggest that costs are indeed resource-dependent. However, a pre-emptive response to future conditions seems unlikely in *Colpidium* due to its short generation time. Rotifers and *Daphnia*, in contrast, do indeed react to high conspecifics’ densities in the so-called crowding response (see above), and this is generally interpreted as an adaptation to future habitat deterioration^15^ (for a similar phenomenon in plants, see^31^). However, as noted above, none of the typical crowding effects were observed in our experiments, nor was any effect of prey density in the absence of predators. It is not even clear to what degree costs in these species depend on resource supply. In *Brachionus*, costs of elongated spines proved difficult to measure, and no clear picture emerges with respect to the effect of population density or food availability^32^. The large crest of induced *Daphnia longicephala* arguably incurs a significant allocation cost^33^, which might be affected by resource availability. In other cases, allocation costs in *Daphnia* have proved difficult to find, and it is more likely that several different types of costs enter into the overall trade-off^34^. For example, in *Daphnia pulex*, defense formation is virtually unaffected by food limitation^28^,^35^, which is a strong argument against density- or resource-dependent costs.

In summary, evidence for density-dependent costs as potential explanation is unlikely in our systems (even considering the possibility of prey anticipating food shortage), whereas density-dependent benefits (i.e., the dilution effect) are likely to be a general phenomenon. We therefore suggest that predation risk assessment plays an important role in the evolution of inducible defenses, and that it might be a general explanation for prey-density-dependent defense expression.

In addition to predator-induced defenses, several organisms have been shown to possess traits directly induced by the presence of competitors. Some of these traits are similar to inducible defenses and are mainly employed in interference competition, such as sweeper tentacles in corals^36^ or spines in rotifers^32^. Others are traded off against investment in defense (e.g. in plants^29^,^31^). Yet others go into the opposite direction of predator-induced defenses. For example, wood frog tadpoles (*Rana sylvatica*) reduce foraging activity and develop deeper tails and shorter bodies in the presence of predators, whereas they develop the opposite suite of traits in the presence of competitors or under food limitation^30^,^37^. When both predators and competitors are present, predator-induced changes are strongest at low competitor density, whereas competitor-induced responses are strongest at low predator-density^25^,^26^,^30^. If competitors are conspecifics, the latter result can be seen as another example of density-dependent reduction of an inducible defense. The reason for this reduction seems to be a trade-off between predation resistance and competitive ability: In addition to their reduced activity, predator-induced tadpoles have smaller mouth parts^26^ and shorter guts (reducing nutrient absorption)^25^, which makes them less efficient foragers and, therefore, poor competitors. In terms of our above discussion, the reduced competitive ability is a density-dependent cost of the inducible defense. An important difference between this system and our study is, however, that we did not observe any phenotypic effects of conspecifics (i.e., intraspecific competitors) in the absence of predators, even though both rotifers^38^ and *Daphnia*^15^,^39^ are known to show life-history responses to strong crowding (see above). This suggests that the inducible defenses studied here do not involve a clear trade-off with competition.

We have here shown that prey use multiple sources of information to determine their investment in anti-predator defenses. In particular, defense expression increases with predator density and decreases with prey density. In the past, effects of prey density have often been ignored in studies of inducible defenses, and our results suggest that this may explain frequently observed and otherwise puzzling differences between the outcomes of different experiments (both within and between studies)^35^. Our study shows that, since conspecifics’ density modifies the degree of defense formation, it needs to be carefully controlled in experiments.

Finally, density-dependent defense expression is likely to have important effects beyond the level of individual fitness, and responses to conspecifics’ density should be incorporated into studies of trait-mediated indirect effects in population and community ecology^40^,^41^.

## Methods

### Study systems

We used six prey species with morphological inducible defenses that can be induced by predator chemicals (kairomones) and that can be accurately quantified: The ciliate *Colpidium kleini* changes from a cylindrical to a spherical shape in response to the predatory ciliate *Lembadion*^11^; the rotifer *Brachionus calyciflorus* develops posterolateral spines in the presence of the predatory rotifer *Asplanchna*^42^; the planktonic crustaceans *Daphnia cucullata* and *D. pulex* produce helmets and ‘neck teeth’, respectively, in response to larvae of the phantom midge *Chaoborus*^43^,^44^ *D. longicephala* forms crests against the backswimmer *Notonecta*^45^; and *D. lumholtzi* develop high helmets in response to fish cues^46^. In all of these systems, the morphological traits have been shown to act as defenses. Their protective effect is correlated to the magnitude of trait expression, which in turn depends on the concentration of the kairomones and, hence, on predator density. Although the trait values used in our study show the magnitude of induction and are related to the protective effect, they are not a direct measure of the protective effect. The real protective effect would be influenced by absolute sizes and behavior. In all six systems, the morphological reaction norms (defense expression versus kairomone concentration) follow saturation curves (e.g.^47^). Finally, all predators in our study show type II (saturating) functional responses^1^,^17^,^18^, such that higher prey density indeed reduces individual predation risk.

### Design of experiments

#### Experiment 1: Effect of predator and prey density on defense expression

To investigate the effects of predator and prey density on the magnitude of defense formation, we used a two-by-two factorial design with two predator and two prey densities (except for the rotifer system, where we only tested a single predator density). We avoided potential pitfalls by only using kairomones for induction to preclude direct selection by predation, by adding antibiotics to prevent differential bacterial breakdown of the kairomones between prey density treatments, and by providing unlimited food concentrations to exclude any food (i.e., competition) effects (see below for a slightly different protocol used in the ciliate system). Preliminary experiments established that prey density had no effect on morphology in the absence of predators.

#### Experiment 2a: Nature of the cue used by *D. lumholtzi*

We used the *D. lumholtzi* system to gain further insight into how prey measures the density of conspecifics. In principle, conspecifics’ density could be measured via a chemical or mechanical cue^48^. To distinguish between these alternatives we again raised prey at two different densities (applying only the high kairomone concentration), and additionally, at low density but in (1 μm filtered) medium transferred from a high prey-density culture.

#### Experiment 2b: Species-specificity of the cue used by *D. lumholtzi*

In situations where conspecifics density is low relative to the density of alternative prey species, it might be advantageous for prey to respond to the combined density of all prey species rather than to only that of its own species. To test for species-specificity of the inducing cue we again raised *Daphnia lumholtzi* in the high-predator treatment at low and high conspecific densities, as well as in a low conspecifics density but together with *Daphnia magna* at a high density.

### Experimental procedures

#### Origin of cultures

*Daphnia cucullata* used in our experiments originated from Lake Thalersee (Bavaria, Germany), *D. pulex* from a pond in Canada, *D. longicephala* from Lara pond (Australia), and *D. lumholtzi* from Fairfield reservoir (Texas, USA). Cultures of *Colpidium kleini* and *Lembadium bullinum* originated from Poland. *Brachionus rubens* and *Asplanchna brightwelli* were isolated from a pond near Ismanning (Bavaria, Germany). All prey organisms coexist in their native habitat with the types of predators employed in our study.

#### Experimental conditions

All experiments were conducted with adequate, naturally occurring prey concentrations (see below). We had verified in control experiments that the prey densities and food effects alone did not induce defenses. To avoid possible influences of food concentrations on defense induction, we fed all prey organisms at non-limiting food concentrations (e.g., *Daphnia cucullata* and *D. pulex* at 1.5, *D. lumholtzi* and *D. longicephala* at 2.5 and rotifers at 1 mg C/L of *Scenedesmus obliquus*) or, in the ciliate system, we did not provide any food at all. All treatments were replicated 10 times, unless stated otherwise. Prior experiments, in which food amount was proportional to prey density, yielded qualitatively similar results. Unless otherwise specified, all experiments were conducted in artificial medium^47^ to exclude natural chemical signals, at 20 ± 0.5 °C in a temperature-controlled room under fluorescent light (16:8 light-dark photoperiod).

#### Kairomones

In the *Daphnia* and rotifer experiments, we used predator kairomones (as opposed to direct contact with predators) for inducing prey defenses, in order to prevent any density-dependent direct selection from predation. To provide constant quality, kairomones were prepared prior to the experiments (by rearing high numbers of predators for 24 h in medium), 0.45-μm filtered, pooled, frozen at −20 ° C in portions for each day, and diluted for use in the experiments. Based on dose-response curves, kairomone concentrations were chosen such that the low concentration induced a weak but significant induction and the high concentration was close to the plateau of the reaction norm. This enabled us to detect response modifications under both conditions. As it is unavoidable in most lab experiments with kairomones, the simulated predator densities were higher than under natural conditions^1^. During experiments, half of the medium was replaced daily to provide constant kairomone and relatively constant conspecific concentrations. Because a higher Daphnia density could lead to a higher bacteria concentration we added in all systems, the antibiotic ampicillin (10 mg/L; Roth, Germany) to avoid possible density-dependent breakdown of the kairomones, after test experiments had proven that the organisms and the induction are not affected.

### *Daphnia* experiments

In all *Daphnia* systems, we started with a single mother and reared age-synchronized cohorts to obtain experimental mothers. The mothers were transferred to the treatments when they carried their first brood, and their third brood (in the treatment) was used for the experiments. Thus, the complete development of the experimental animals took place in the specific treatment. Experimental animals were selected randomly within the treatments.

In the *D. lumholtzi* system, we reared 1 *Daphnia* in 200 mL medium for the low and 10 daphnids for the high prey-density treatment. *Daphnia* were measured when they reached maturity. Fish kairomone concentration was added at a concentration of 1 stickleback (*Gasterosteus aculeatus*, 3.5 cm)/(L × day) in the high and 0.05 stickleback/(L × day) in the low-predator treatment.

For the experiments displayed in Fig. 2 (nature and specificity of the cue used by *D. lumholtzi*), we produced new batches of kairomone and only used the high kairomone concentration. For Fig. 2a (chemical vs. mechanical cue), we first repeated the experiments as described above with both high and low prey densities (the high-density treatment had only 9 replicates). Additionally, we placed *D. lumholtzi* at low density into 1-μm filtered medium from a high-density culture, allowing only chemical cues to act. For Fig. 2b (species-specificity of the cue), we again repeated the high and low prey-density experiments, and additionally placed *D. lumholtzi* at low density together with a high density of *D. magna* (1 *D. lumholtzi* reared together with 9 *D. magna*).

In the *D. pulex* system, we reared 2 daphnids in 200 mL medium for the low and 20 for the high prey density. We measured and scored individual neck teeth in the second juvenile stage, when defense formation is strongest in this clone^47^. As the *Chaoborus flavicans* larvae were just in the second stage and very small compared to fourth instar larvae, we used kairomone concentrations corresponding to 20 larvae/(L × day) in the low and 200/(L × day) in the high predator treatment.

In the *D. longicephala* system, we reared 1 *Daphnia* in 250 mL medium for the low and 10 for the high prey density treatment. Kairomone concentrations corresponded to 0.75 *Notonecta glauca*/(L × day) in the high and 0.075 *N. glauca*/(L × day) in the low predator density treatment.

For the small *D. cucullata*, we used 10 individuals in 200 mL medium as low and 25 individuals as high prey concentration. The *Chaoborus flavicans* kairomone concentration was 10 fourth-instar larvae/(L × day) for the low and 25/(L × day) for the high-density treatment.

The reason we used different prey densities across the Daphnia experiments was an attempt to account for the size differences between the various species.

### Rotifer experiments

The rotifer experiments were conducted in 2 mL microtiter plates (which allowed exact control over the predator and prey densities) in an incubator at 15 °C in the dark. The low prey density was 2 *Brachionus* (34 replicates), the high density 20 *Brachionus* (10 replicates). We used *Asplanchna* kairomone in a concentration that had induced intermediate spines in preliminary experiments. Because defenses in *B. calyciflorus* are induced via the mother generation^42^, we reared F0- and F1-generations under experimental conditions and measured the F1-generation at the age of 5 days.

### Protozoa experiments

Induction of *Colpidium kleini* by *Lembadion bullinum* was studied with two prey densities (200 and 2000 per mL) and two predator densities (20 and 200 per mL) in 10 mL medium at 20 °C in the dark (5 replicates per treatment). Although it is possible to induce *Colpidium* with *Lembadion*-conditioned medium^49^, the resulting transformation is weak. Therefore, we kept both species in direct contact to each other. To minimize predation, *Lembadion* were starved for several days before the experiment. This resulted in extremely small cells, which were poorly able to ingest their prey, and based on observed feeding rates, it can be excluded that the reported effects have been caused by selective predation. No food was offered to *Colpidium* during the experiments^12^. The experimental animals were fixed with glutaraldehyde after 48 h. Lengths and widths of *Colpidium* were measured, and the ratio was used as an index of the degree of defense.

### Measurements and analysis

All measurements were conducted using a digital image analysis system (AnalySIS, Soft Imaging Systems, Münster, Germany). Statistics were calculated with SPSS (V11.5). Ratio values were arcsin square root transformed or log transformed (ciliates) before analysis. After transformation, all data, except those from the rotifer experiment, followed a normal distribution with homogeneous variances. The rotifer experiment was, therefore, analyzed with a non-parametric Mann Whitney U-test. All other results were analyzed by means of two-way ANOVAs with the factors prey density and predator density.

## Additional Information

**How to cite this article**: Tollrian, R. *et al*. Density-dependent adjustment of inducible defenses. *Sci. Rep.* **5**, 12736; doi: 10.1038/srep12736 (2015).

## References

[b1] Tollrian R. & Harvell C. D. The ecology and evolution of inducible defenses. (Princeton University Press 1999).

[b2] Kats L. B. & Dill L. M. The scent of death: chemosensory assessment of predation risk by prey animals. Ecoscience. 5, 361–394 (1998).

[b3] Bertram B. C. R. Living in groups: predators and prey in Behavioural Ecology: an Evolutionary Approach. (ed. Krebs J. R. & Davies N. B. ) pp. 64–96 (Blackwell Scientific, Oxford 1978).

[b4] Hamilton W. D. Geometry for the selfish herd. J Theor Biol. 31, 295–311 (1971). 5104951 10.1016/0022-5193(71)90189-5

[b5] Peacor S. D. Phenotypic modifications to conspecific density arising from predation risk assessment. Oikos. 100, 409–415 (2003).

[b6] Jeschke J. M. Density-dependent effects of prey defenses and predator offenses. J Theor Biol. 242, 900–907 (2006). 16842823 10.1016/j.jtbi.2006.05.017

[b7] Ale S. B., Brown J. S. The contingencies of group size and vigilance. Evol Ecol Res. 9, 1263–1276 (2007).

[b8] Hanazato T. Influence of food density on the effects of a *Chaoborus*-released chemical on Daphnia ambigua. Freshw Biol. 25, 477–483 (1991).

[b9] Anholt B. R. & Werner E. E. Interaction between food availability and predation mortality mediated by adaptive behavior. Ecology. 76, 2230–2234 (1995).

[b10] Pettersson L. B. & Brönmark C. Density-dependent costs of an inducible morphological defense in crucian carp. Ecology. 78, 1805–1815 (1997).

[b11] Fyda J. & Wiackowski K. Benefits and costs of predator-induced morphological changes in the ciliate Colpidium kleini (Protozoa, Ciliophora). Europ J of Protozool. 34, 118–123 (1998).

[b12] Wiackowski K. & Starońska A. The effect of predator and prey density on the induced defence of a ciliate. Funct Ecol. 13, 59–65 (1999).

[b13] McCoy M. W., Conspecific density determines the magnitude and character of predator-induced phenotype. Oecologia. 153, 871–878 (2007). 17636335 10.1007/s00442-007-0795-y

[b14] Van Buskirk J., Ferrari M., Kueng D., Näpflin K. & Ritter N. Prey risk assessment depends on conspecific density. Oikos. 120, 1235–1239 (2011).

[b15] Burns C. W. Crowding-induced changes in growth, reproduction and morphology of *Daphnia*. Freshw Biol. 43, 19–29 (2000).

[b16] Jeschke J. M., Kopp M. & Tollrian R. Predator functional responses: discriminating between handling and digesting prey. Ecolog Monogr. 72, 95–112 (2002).

[b17] Jeschke J. M., Kopp M. & Tollrian R. Consumer-food systems: why type I functional responses are exclusive to filter feeders. Biol Rev Camb Philos Soc. 79, 337–349 (2004). 15191227 10.1017/s1464793103006286

[b18] Jeschke J. M. & Tollrian R. Density-dependent effects of prey defences. Oecologia. 123, 391–396 (2000). 10.1007/s004420051026 28308594

[b19] Stankowich T. & Blumstein D. T. Fear in animals: a meta-analysis and review of risk assessment. Proc R Soc B. 272, 2627–2634 (2005). 10.1098/rspb.2005.3251 PMC1559976 16321785

[b20] Laforsch C., Beccara L. & Tollrian R. Inducible defenses: The relevance of chemical alarm cues in Daphnia. Limnol Oceanogr. 51, 1466–1472 (2006).

[b21] Wilson K., Thomas M. B., Blanford S., Doggett M., Simpson S. J. & Moore S. L. Coping with crowds: density-dependent disease resistance in desert locusts. Proc Natl Acad Sci. 99, 5471–5475 (2002). 11960003 10.1073/pnas.082461999 PMC122793

[b22] Elliot S. L. & Hart A. G. Density-dependent prophylactic immunity reconsidered in the light of host group living and social behavior. Ecology. 91, 65–72 (2010). 20380197 10.1890/09-0424.1

[b23] Yin M., Laforsch C., Lohr J. N. & Wolinska J. Predator-induced defense makes *Daphnia* more vulnerable to parasites. Evolution. 65, 1482–1488 (2011). 21521197 10.1111/j.1558-5646.2011.01240.x

[b24] Tollrian R. & Harvell D. H. The evolution of inducible defenses: current ideas in the ecology and evolution of inducible defenses. (eds Tollrian R., Harvell D. C. ). pp. 307–321 (Princeton University Press 1999).

[b25] Relyea R. A. & Auld J. R. Having the guts to compete: how intestinal plasticity explains costs of inducible defences. Ecol Lett. 7, 869–875 (2004).

[b26] Relyea R. A. & Auld J. R. Predator-and competitor-induced plasticity: how changes in foraging morphology affect phenotypic trade-offs. Ecology. 86, 1723–1729 (2005).

[b27] Wiackowski K. & Szkarlat M. Effects of food availability on predator-induced morphological defence in the ciliate *Euplotes octocarinatus* (Protista). Hydrobiol. 321, 47–52 (1996).

[b28] Spaak P. & Boersma M. Tail spine length in the *Daphnia galeata* complex: costs and benefits of induction by fish. Aqu Ecol. 31, 89–98 (1997).

[b29] Cipollini D. Stretching the limits of plasticity: can a plant defend against both competitors and herbivores? Ecology. 85, 28–37 (2004).

[b30] Relyea R. A. F.ine-tuned phenotypes: tadpole plasticity under 16 combinations of predators and competitors. Ecology. 85, 172–179 (2004).

[b31] Izaguirre M. M., Mazza C. A., Biondini M., Baldwin I. T. & Ballaré C. L. Remote sensing of future competitors: impacts on plant defenses. Proc Natl Acad Sci. 103, 7170–7174 (2006). 16632610 10.1073/pnas.0509805103 PMC1459035

[b32] Gilbert J. Kairomone induced morphological changes in rotifers in the ecology and evolution of inducible defenses. (eds Tollrian R. & Harvell D. C. ) pp. 177–202 (Princeton University Press 1999).

[b33] Barry M. J. The costs of crest induction for *Daphnia carinata*. Oecologia. 97, 278–288 (1994). 10.1007/BF00323161 28313940

[b34] Tollrian R., Dodson S. D. Inducible defenses in Cladocera: Constraints, Costs and multipredator environments in the ecology and evolution of inducible defenses. (eds Tollrian R. & Harvell D. C. ) pp.177–202 (Princeton University Press 1999).

[b35] Tollrian R. Predator-induced morphological defenses: costs, life history shifts, and maternal effects in *Daphnia pulex*. Ecology. 76, 1691–1705 (1995).

[b36] Chornesky E. A. Induced development of sweeper tentacles on the reef coral *Agaricia agaricites*: a response to direct competition. Biol Bull. 165, 569–581 (1983). 10.2307/1541466 29324010

[b37] Relyea R. A. Competitor-induced plasticity in tadpoles: consequences, cues, and connections to predator-induced plasticity. Ecol Monogr. 72, 523–540 (2002).

[b38] Gilbert J. J. Specificity of crowding response that induces sexuality in the rotifer *Brachionus*. Limnol Oceanogr. 48, 1297–1303 (2003).

[b39] Lürling M. The effect of substances from different zooplankton species and fish on the induction of defensive morphology in the green alga *Scenedesmus obliquus*. J Plankton Res. 25, 979 (2003).

[b40] Werner E. E. & Peacor S. D. A review of trait-mediated indirect interactions in ecological communities. Ecology. 84, 1083–1100 (2003).

[b41] Miner B. G., Sultan S. E., Morgan S. G., Padilla D. K. & Relyea R. A. Ecological consequences of phenotypic plasticity. Trends Ecol Evol. 20, 685–692 (2005). 16701458 10.1016/j.tree.2005.08.002

[b42] Gilbert J. J. Rotifer ecology and embryological induction. Science. 151, 1234–1237 (1966). 5910006 10.1126/science.151.3715.1234

[b43] Tollrian R. Predator-induced helmet formation in *Daphnia cucullata* (Sars). Arch f Hydrobiol. 119, 191–196 (1990).

[b44] Krueger D. A. & Dodson S. I. Embryological induction and predation ecology in *Daphnia pulex*. Limnol and Oceanogr. 26, 219–223 (1981).

[b45] Grant J. W. G. & Bayly I. A. E. Predator induction of crests in morphs of the *Daphnia carinata* King Complex. Limnol and Oceanogr. 26, 201–218 (1981).

[b46] Tollrian R. Fish-kairomone induced morphological changes in *Daphnia lumholtzi* (Sars). Arch f Hydrobiol. 130, 69–69 (1994).

[b47] Tollrian R. Neckteeth formation in *Daphnia pulex* as an example of continuous phenotypic plasticity: morphological effects of *Chaoborus* kairomone concentration and their quantification. J Plankton Res. 15, 1309–1318 (1993).

[b48] Laforsch C. & Tollrian R. Cyclomorphosis and phenotypic defences in Encyclopedia of Inland waters (ed Edited by Liekens G. E. ) Vol. 3 pp. 643–650 (Elsevier, 2009).

[b49] Fyda J. Predator-induced morphological changes in the ciliate *Colpidium* (Protozoa, Ciliophora). *Europ* J Protozool. 34, 111–117 (1998).

